# The interplay between WASH practices and vaccination with oral cholera vaccines in protecting against cholera in urban Bangladesh: Reanalysis of a cluster-randomized trial

**DOI:** 10.1016/j.vaccine.2023.02.054

**Published:** 2023-03-31

**Authors:** Fahima Chowdhury, Asma Binte Aziz, Faisal Ahmmed, Tasnuva Ahmed, Sophie SY Kang, Justin Im, Juyeon Park, Birkneh Tilahun Tadesse, Md. Taufiqul Islam, Deok Ryun Kim, Masuma Hoque, Gideok Pak, Farhana Khanam, Nigel A.J. McMillan, Xinxue Liu, Khalequ Zaman, Ashraful Islam Khan, Jerome H. Kim, Florian Marks, Firdausi Qadri, John D. Clemens

**Affiliations:** a International Centre for Diarrheal Disease Research, Bangladesh, Dhaka, Bangladesh; b International Vaccine Institute, Seoul, Republic of Korea; c Oxford Vaccine Group, Department of Pediatrics, University of Oxford, Oxford OX3 9DU, United Kingdom; d Department of Medicine, University of Cambridge, Cambridge, United Kingdom; e University of Antananarivo, Antananarivo, Madagascar; f UCLA Fielding School of Public Health, Los Angeles, CA 90095-1772, USA; g Menzies Health Institute Queensland, Griffith University, Gold Coast, Australia

**Keywords:** Cholera, WASH, Oral cholera vaccine, Cluster randomized trials, Vaccine effectiveness

## Abstract

The current global initiative to end Cholera by 2030 emphasizes the use of oral cholera vaccine (OCV) combined with feasible household Water-Sanitation-Hygiene (WASH) interventions. However, little is known about how improved WASH practices and behaviors and OCV interact to reduce the risk of cholera.

We reanalyzed two arms of a cluster-randomized trial in urban Bangladesh, to evaluate the effectiveness of OCV given as a 2-dose regimen. One arm (30 clusters, n = 94,675) was randomized to vaccination of persons aged one year and older with OCV, and the other arm (30 clusters, n = 80,056) to no intervention. We evaluated the prevention of cholera by household WASH, classified at baseline using a previously validated rule, and OCV over 2 years of follow-up. When analyzed by assignment to OCV clusters rather than receipt of OCV, in comparison to persons living in “Not Better WASH” households in the control clusters, reduction of severe cholera (the primary outcome) was similar for persons in “Not Better WASH” households in vaccine clusters (46%, 95% CI:24,62), for persons in “Better WASH” households in the control clusters (48%, 95% CI:25,64), and for persons in “Better WASH” households in the vaccine clusters (48%, 95% CI:16,67). In contrast, when analyzed by actual receipt of a complete OCV regimen, , in comparison to persons in “Not Better WASH” households in the control clusters, protection against severe cholera increased steadily from 39% (95% CI:13,58) in residents of “Better WASH” households in the control clusters to 57% (95% CI:35,72) in vaccinated persons in “Not Better WASH” households to 63% (95% CI:21,83) in vaccinated persons in “Better WASH” households.

This analysis suggests that improved household WASH and OCV received may interact to provide greater protection against cholera. However, the divergence between findings related to intent to vaccinate versus those pertaining to actual receipt of OCV underscores the need for further research on this topic.

## 1 Introduction

Both endemic and epidemic cholera remain major public health problems in many low and middle-income countries (LMICs), with an estimated death toll nearing 100,000 deaths annually [1]. Despite decades of global recommendations to implement improved water, sanitation, and hygiene (WASH), major municipal WASH infrastructural improvements, which have eliminated cholera as a public health problem in affluent countries, have proven to be too expensive to be feasible in the near term, and the global incidence of cholera has not declined [2]. Inexpensive, inactivated oral cholera vaccines (OCVs), which have been shown to be safe and effective for at least five years after dosing, have been recommended by the World Health Organization (WHO) for use in both endemic and epidemic settings since 2011 [3], [4]. In recognition of the public health value of these OCVs, a global OCV stockpile for use in cholera epidemics, cholera endemic settings, and humanitarian emergencies was established in 2013 by WHO, with financial support from Gavi [5]. Since the establishment of the stockpile, more than 100 million doses have been distributed, with the number nearly doubling each year prior to the COVID pandemic, and with demand by countries regularly far exceeding supply [6]. As well, evaluations of OCV deployments from the stockpile have consistently found the vaccines to be well accepted, feasibly delivered, and highly protective against cholera in real-life settings [7], [8], [9].

The success of the stockpile, together with the persistently high burden of cholera globally, helped catalyze a major global initiative led by WHO in 2017 entitled “Ending Cholera: The Global Roadmap to 2030,” with the ambitious goal of dramatically reducing cholera cases and deaths in endemic settings [9]. The major pillars of the initiative are to intensify surveillance for cholera, to deploy inactivated OCVs, and to implement feasible improvements in WASH through multisectoral programs in cholera hotspots in LMICs affected by endemic cholera [9]. Although the success of the initiative rests in part on how inactivated OCVs and modest improvements in WASH will work together to prevent cholera, studies in the field to evaluate this potential interaction are sparse.

We conducted a three-armed cluster-randomized, controlled trial (CRT) in 2011 in an urban slum of Dhaka, Bangladesh, to assess the protection of the inactivated OCV, Shanchol^TM^, (Shantha Biotechnics, Hyderabad, India) delivered to persons aged one year and older under realistic public health conditions and the extent to which a simple, low-cost intervention, conducted at the household level and designed to improve use of clean water and appropriate handwashing practices, augmented the protection by OCV [10]. The trial, which compared arms of clusters randomized to OCV alone, OCV combined with the WASH intervention, or no intervention, found that although OCV made a significant impact on the occurrence of severe cholera, the primary outcome for the trial, the WASH intervention failed to augment the protection by OCV [10]. However, because the population uptake of the WASH intervention was poor, the trial did not rule out the possible enhancement of OCV protection by an effective WASH intervention.

Earlier work in slums of urban Bangladesh had demonstrated that natural variations in WASH behaviors and practices were strongly associated with the risk of pediatric diarrhea [11]. We therefore evaluated how WASH behaviors and practices that varied naturally in households of the control clusters of the CRT predicted the risk of severe cholera in members of the households. The analysis derived and validated a rule for WASH behaviors and practices, dichotomizing households as “Better” and “Not Better” WASH, and found that members of households with “Better WASH” had a notably lower risk of severe cholera [17] . In this paper, we use this WASH classification to examine in the CRT how “Better WASH” in the household, ascertained at baseline in the trial, interacted with OCV to protect against cholera.

## 2 Methods

### 2.1 The cluster-randomized trial of OCV and WASH

As described in detail elsewhere (9), the CRT was conducted in a densely populated urban slum in Mirpur, Dhaka. Participants were residents of households of the slum considered at higher risk of cholera by *a priori* criteria related to lower socioeconomic status and poor access to good sanitation and unhygienic living conditions. A baseline census was conducted from April-September 2010, before the mass vaccination campaign with the 2-dose regimens of the OCV, Shanchol™, assigning a unique study identification numbers which were used for both demographic and clinical surveillance. The census was updated at 6-month intervals for births, deaths, and migrations thereafter. The baseline census questionnaire contained several questions about household water sources, water storage and use, sanitation facilities available, and routine handwashing practices. In total, 268,896 persons were enumerated in the baseline study population.

At baseline, the study area was demarcated into 90 geographical clusters with approximately the same population sizes; each cluster was separated circumferentially from adjacent clusters by a buffer zone approximately 30 m wide, intended to minimize spread of WASH messages between clusters. Prior to the OCV campaign, the clusters were block-randomized within strata defined by distance of clusters to the nearest treatment center, to one of three arms, each with 30 clusters,: an arm in which OCV was targeted to non-pregnant persons aged one year and older but no WASH intervention; an arm in which OCV was targeted to persons fulfilling same criteria and all members were targeted for the WASH intervention; and a control arm in which no intervention was delivered. Surveillance for cholera was instituted at twelve hospitals serving the study area, in which patients from the study area were identified by either census identification numbers or, if such cards were not available, identification numbers from a computerized census available at each treatment site. These unique identification numbers were used to link treatment visits to the census and thus to the identity of the treatment arm in which the patients belonged. After obtaining informed consent, physicians carried out a clinical examination and obtained fecal specimens. All fecal specimens were evaluated for *Vibrio cholerae (V. cholerae)* as previously described [10]. For patients from whom a fecal culture yielded *V. cholerae* O1 or O139, a visit was made to the home of visit, ascertained upon presentation, within 7 days of discharge to confirm the identity of the person whose name had been given at the treatment center.

### 2.2 Definitions

A treatment center visit for diarrhea was considered to be any visit in which a patient reported having 3 or more loose stools or 1–2 or an indeterminate number of loose stools with evidence of dehydration in the 24 h before presentation. If the date of discharge from an earlier diarrheal visit and the date of symptom onset for the subsequent diarrheal visit were within 7 days of one another, both visits were considered part of the same diarrheal episode. The onset of a diarrheal episode was defined as starting when the patient first reported loose or liquid stools prior to the first diarrhoeal visit of the episode. A cholera episode was defined as a diarrheal episode in which a fecal specimen from any constituent visit yielded V. cholerae O1 or O139 and where bloody stool was not reported. Severely dehydrating cholera, defined by WHO criteria, was defined when the patient exhibited severe dehydration during any visit of the episode [12]. In order for an episode to be analyzed as cholera, we also required that the visit made to the patient’s home after isolation of V. cholerae O1 or O139 confirmed that the person whose identity had been given at the treatment center had indeed sought treatment for diarrhea on the date of presentation.

In the same trial population, we developed a dichotomous variable that classified households as having “Better WASH” or “Not Better WASH” at baseline and that was predictive of the ensuing risk of severe cholera [17]. This composite variable was constructed from WASH characteristics recorded during the baseline census. Using a derivation subpopulation of a random sample of 50% of households in the control clusters, we applied recursive partitioning (a machine learning algorithm) to select and conjoin household WASH variables that were most predictive of risk of severe cholera during the subsequent four years of follow-up. With this analysis, we defined a “Better WASH” household as one in which drinking water was pretreated by filtering, boiling, or appropriate chemicals, and in which the drinking water source was located nearby the house; or if the drinking water sources were not near the house, pretreatment of drinking water was used and the source of drinking water was private tap, well, pump, bottled water or water from the vendor. Households not fulfilling these criteria were classified as having Not Better WASH. We then confirmed that this rule had nearly identical levels of sensitivity and specificity for predicting the risk of severe cholera in a validation subpopulation consisting of the other 50% of the households in the control clusters.

### 2.3 Analysis

To evaluate the interplay between preexisting household WASH and OCV in preventing all episodes of cholera as well as severe cholera, we included only the OCV-only and control clusters of the trial since the OCV-WASH clusters had received an external WASH intervention. We approached the problem in two ways, following our original analysis of the trial [10]. Firstly, we considered the population analyzed for overall OCV protection, comparing the incidence of cholera in all residents of OCV-only clusters versus all persons in the control clusters, stratifying households according to whether they had “Better WASH” or “Not Better WASH” at baseline. This analysis thus evaluated the impact of a program of administering OCV in households that had or did not have “Better WASH”. Secondly, we evaluated the population analyzed for total OCV protection*,* comparing the incidence of cholera in residents of OCV clusters who received two complete doses of OCV versus persons age-eligible for OCV residing in the control clusters, again stratifying by residence in a household with “Better WASH” or “Not Better WASH” at baseline. This analysis provided a different perspective, evaluating the protection against cholera owing to receipt of OCV, depending on whether or not a subject resided in a household with “Better WASH”.

We analyzed the fixed cohort present at the time of OCV dosing. Following our original analysis of the trial (9), we defined the start date of follow-up 14 days after the second dose of OCV, the median date of 14 days after the second dose in OCV clusters for residents who did not receive two doses of the vaccine, and 14 days after the median date of second dose of OCV in the nearest OCV cluster for residents of the control clusters. Follow-up of subjects was censored at migration out of the cluster, death, or two years of follow-up, whichever came first. Two years was selected as the maximum duration of follow-up because the population was highly mobile (ca. 25% migration per year), and a low fraction of the baseline cohort was still under follow-up thereafter.

To evaluate protective effectiveness (PE), we used survival analysis. We fitted unadjusted and adjusted Cox proportional hazards regression models after verifying that the proportionality assumptions were fulfilled for independent variables. We then estimated the hazard ratios by exponentiating the coefficient of the group variable in these models. PE was calculated as (1–hazard ratio) × 100%, and robust sandwich variance estimates were used to account for the design effect of cluster randomization, allowing inferences for vaccine protection at the individual level. Several baseline variables were considered as potential confounders for inclusion as independent variables in the models: age, gender, religion, average household expenditure, residence in a house owned by the family, residence in a household with only one room, residence in a household with a concrete roof, a history of diarrhea within the previous 6 months, residence in the study area for<1 year, and residence in a household that knew about cholera vaccine. After forcing the variable for stratified randomization in the models, other variables were introduced via stepwise backward-elimination, starting with all potential covariates and sequentially removing the covariates until no further increase was observed in the Akaike Information Criterion. Two-way interactions between the OCV and WASH terms in the models were also considered as independent variables. The threshold of significance for individual estimates of protective effectiveness was p < 0.05 with a corresponding 95% CI (two-sided). All statistical analyses were done using R version 4.1.0.

### 2.4 Registration and approvals

The trial is registered at ClinicalTrials.gov number NCT01339845. Ethical approval was obtained from the Ethical Review Committee of the International Centre for Diarrhoeal Disease Research, Bangladesh (icddr,b), and informed consent was obtained from study participants.

## 3 Results

### 3.1 Trial Participants

**Fig. 1:**
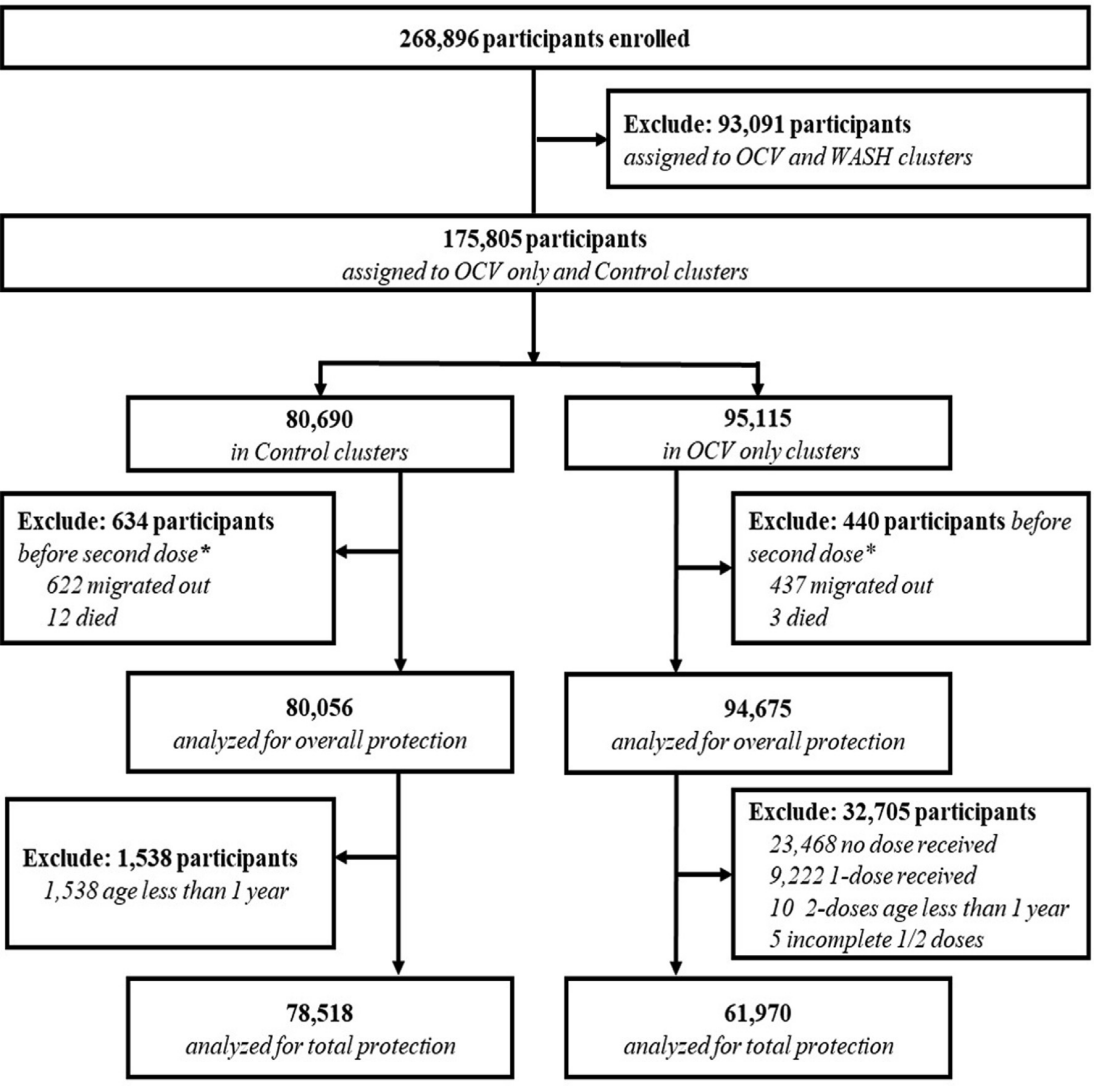
CONSORT diagram for assembly of the overall and total OCV protection. * Median date of second dose for recipients of 1-dose or no dose.

Baseline vaccine coverage with two doses of OCV was 65% (61,970/94,675) in the OCV clusters (Fig. 1). The mean age (standard deviation, SD) of the residents at the baseline was 23.9 (±15.8) years in the OCV clusters and 24.1(±16.0) years in the control clusters for the overall protection analysis; similar ages were found for residents in the OCV clusters and control clusters included in total protection analysis. Other baseline characteristics were also well balanced between the OCV and control arms for the analyses of both overall and total OCV protection; similarly, baseline features were well balanced between households with “Better WASH” and “Not Better WASH” in the populations evaluated for overall and total OCV protection (Table 1 and Supplementary data 1 ).There were 94,675 persons assigned to the OCV clusters, 26% (24,307/94,675) of whom resided in households with Better WASH (Table 2, Table 3). In control clusters, there were 80,056 people, 27% (21,972/80,056) of whom resided in Better WASH households (Table 3). For the analysis of the population assessed for total OCV protection, 61,970 people in OCV clusters and 78,518 in the control clusters were included (Table 4).

**Table 1:** Baseline characteristics of the residents of OCV and control clusters of the trial in the evaluation of overall and total OCV protection.

	Overall Protection			Total Protection		
	Residence in control clusters (n = 80,056)	Residence in OCV clusters (n = 94,675)	p-value	Residence in control clusters (n = 78,518)	Residence in OCV clusters (n = 61,970)	p-value
Mean age (SD*; years)	24.1 ± 16.0	23.9 ± 15.8	0.021	24.6 ± 15.8	23.1 ± 15.8	<0.001
Male participants - n# (%)	39,264 (49.0)	45,677 (48.2)	0.001	38,485 (49.0)	27,899 (45.0)	<0.001
Live in own house - n# (%)	20,075 (25.1)	19,892 (21.0)	<0.001	19,714 (25.1)	14,004 (22.6)	<0.001
Live in a household with only one room - n# (%)	64,679 (80.8)	78,173 (82.6)	<0.001	63,389 (80.7)	50,844 (82.0)	<0.001
Live in a household with a concrete roof- n# (%)	67,532 (84.4)	83,263 (87.9)	<0.001	66,249 (84.4)	54,957 (88.7)	<0.001
Diarrhoea within previous 6 months - n# (%)	11,189 (14.0)	12,657 (13.4)	<0.001	11,050 (14.1)	9014 (14.5)	0.012
Mean time living in the area (SD*; months)	74.1 ± 121.7	67.9 ± 116.9	<0.001	74.3 ± 121.9	74.9 ± 121.9	0.349
Lived in study area for<1 year - n# (%)	32,424 (40.5)	43,174 (45.6)	<0.001	31,725 (40.4)	26,072 (42.1)	<0.001
Live in a household that knows about cholera vaccine - n# (%)	6432 (8.0)	5944 (6.3)	<0.001	6306 (8.0)	2334 (3.8)	<0.001
Mean number of individuals per household (SD*)	4.8 ± 1.9	4.7 ± 2.0	<0.001	4.8 ± 1.9	4.8 ± 1.9	0.441
Median distance to the nearest icddr,b hospital (meters) (IQR^II^)	1802 (994,2414)	1792 (1121,2266)	<0.001	1802 (991,2414)	1777 (1101,2255)	<0.001

* Standard deviation.

^II^ Interquartile range.

# Number of individuals.

### 3.2 Protection against all cholera episodes and severe cholera episodes in the population evaluated for overall OCV protection

A total of 442 confirmed cholera episodes, 171 severe, were diagnosed in the study population assessed for overall OCV protection during the 2 years of the follow-up. Two hundred fifty-nine cholera cases (106 severe) occurred among the residents in control clusters, and 183 cases (65 severe) occurred in OCV clusters (Table 2). Corresponding incidence rates (IR) per 100,000 person-years of observation (PYO) in the control and OCV clusters were 241 and 160 for all cholera episodes, and 98 and 57 for severe cholera episodes, respectively. The adjusted overall PE associated with residing in the OCV clusters, after controlling for the WASH status of the subject’s household and other potential confounders, was 31% (95% confidence interval (CI):12, 46) for all cholera episodes and 39% (95% CI:17, 56) for severe cholera episodes (Table 2). Similarly, 343 cases of cholera and 139 severe cholera were diagnosed among residents of “Not Better WASH” households, compared to 99 cases of cholera and 32 severe cholera in residents of “Better WASH” households. Corresponding IRs per 100,000 PYO in “Not Better WASH” and “Better WASH” households were 211 and 167 for all cholera episodes, and 85 and 54 cases for severe cholera episodes. Overall protection associated with living in a “Better WASH” household was 26% (95% CI: −2, 46) against all cholera episodes and 32% (95% CI; 6, 51) against severe cholera episodes after controlling for residence in an OCV versus control cluster and other relevant confounders (Table 2).

**Table 2:** Protection against all cholera and severe cholera episodes in residents of OCV versus control clusters or by residence in Better or Not Better WASH households in the population evaluated for overall OCV protection.

	Participants (n)	Case episodes;100000PY*	Incidence (Cases per 100,000 PY*; 95%CI)	p-value I (Trend test)	Crude PE#	p-value	Adjusted PE#	p-value
All cholera								
Residence in control clusters	80,056	259;107671	241 (211,270)		Ref.	–	Ref.	–
Residence in OCV clusters	94,675	183;114536	160 (137,183)	<0.001	34 (12,50)	0.005	31(12,46)¶	0.004
All cholera								
Residence in Not Better WASH households	128,452	343;162829	211 (188,233)		Ref.	–	Ref.	–
Residence in Better WASH households	46,279	99;59378	167 (134,200)	0.040	21 (-6,41)	0.118	26(-2,46) §	0.063
Severe cholera								
Residence in control clusters	80,056	106;107773	98 (80,117)		Ref.	–	Ref.	–
Residence in OCV clusters	94,675	65;114594	57 (43,71)	<0.001	42 (17,60)	0.003	39(17,56) ¶	0.002
Severe cholera								
Residence in Not Better WASH households	128,452	139;162943	85 (71,99)		Ref.	–	Ref.	–
Residence in Better WASH households	46,279	32;59424	54 (35,73)	0.018	37 (11,56)	0.010	32(6,51) §	0.020

* Person-years.

# Protective effectiveness.

I One-sided p-value calculated using Cochran–Armitage test.

¶ Adjusted for closer distance from the household to the nearest icddr,b hospital, age at zero time (years), household average expenditure, individuals living in their own house, individuals living in a household with only one room, individuals living in a household with a concrete roof, individuals having reported diarrhoea within 6 months at the time of household registration, total number of household members, household wash status.

§ Adjusted for closer distance from the household to the nearest icddr,b hospital, age at zero time (years), household average expenditure, individuals living in their own house, individuals living in a household with only one room, individuals living in a household with a concrete roof, individuals having reported diarrhoea within 6 months at the time of household registration, total number of household members, residence in a vaccine versus control cluster.

We further analyzed the combined overall protective impact of OCV and “Better WASH” against all cholera and severe cholera by the four possible categories of the two variables: residence in a “Not Better WASH” household in a control cluster; residence in a “Better WASH” household in a control cluster; residence in a “Not Better WASH” household in an OCV cluster; and residence in a “Better WASH” household in an OCV cluster. Relative to residence in a “Not Better WASH” household in a control cluster, residence in a “Better WASH” household in an OCV cluster was associated with 41% (95% CI: 13, 59) protection against all cholera episodes and 48% (95% CI: 16, 67) protection against severe cholera episodes (Table 3). Very similar results for protection against all cholera episodes and severe cholera episodes were observed for persons living in “Better WASH” households in control clusters and for, persons living in “Not Better WASH” households in the OCV clusters (Table 3).

### 3.3 Protection against all cholera episodes and severe cholera episodes in the population evaluated for total OCV protection

Because the analysis of overall protection reflected only the assignment to the OCV versus control clusters, rather than the receipt of a 2-dose regimen of OCV, we repeated these analyses for the population considered for total OCV protection, in whom there were 319 cholera episodes, of which 139 were severe (Table 4). Protection associated with receipt of 2 doses of OCV was 40% (95% CI:21, 54) against all cholera episodes and 55% (95% CI: 32, 70) against severe cholera, after controlling for household WASH status and other potentially confounding variables. Protection associated with residence in a “Better WASH” household was 26% (95% CI: 0, 46) and 33% (95% CI: 5, 53) against all cholera episodes and severe cholera episodes, respectively, after controlling for OCV status and other potentially confounding variables (Table 4). Joint consideration of receipt of OCV and of household WASH status revealed that, relative to persons who lived in control clusters and resided in a “Not Better WASH” household, estimates of protection increased from persons living in control clusters and residing in “Better WASH” households was [34% (95% CI: 2, 55)] for all cholera and 39% (95% CI:13,58) for severe cholera, to persons living in “Not Better WASH” households who received OCV [44% (95% CI; 26, 58) for all cholera and 57% (95% CI: 35, 72) for severe cholera], and finally to persons living in “Better WASH” households who received OCV was [49% (95% CI: 21, 67) for all cholera, and 63% (95% CI: 21, 83) for severe cholera episodes] (Table 5). The trend is statistically significant at p-value < 0.05 in all four catagories for all cholera and severe cholera cases.

**Table 3:** Protection against all cholera and severe cholera by assignment to OCV versus control clusters and by residence in Better or Not Better WASH households considered conjointly in the population evaluated for overall OCV protection.

	Participants (n)	Case episodes;100000PY*	Incidence (Cases per 100,000 PY*; 95%CI)	p-value II (Trend test)	Crude PE# (95%CI)	p- value	Adjusted¶ PE# (95%CI)	p-value
All cholera								
Residence in control clusters: Not Better WASH households	58,084	209;78009	268 (232,304)		Ref.	–	Ref.	–
Residence in control clusters: Better WASH households	21,972	50;29662	169 (122,215)		37 (9,57)	0.015	40 (10,60)	0.014
Residence in OCV clusters: Not Better WASH households	70,368	134;84820	158 (131,185)		41 (18,58)	0.002	38 (18,54)	0.001
Residence in OCV clusters: Better WASH households	24,307	49;29716	165 (119,211)	<0.001	38 (5,60)	0.027	41 (13,59)	0.007
Severe cholera								
Residence in control clusters: Not Better WASH households	58,084	90;78086	115 (91,139)		Ref.	–	Ref.	–
Residence in control clusters: Better WASH households	21,972	16;29686	54 (27,80)		53 (30,69)	<0.001	48 (25,64)	<0.001
Residence in OCV clusters: Not Better WASH households	70,368	49;84857	58 (42,74)		50 (25,67)	0.001	46 (24,62)	<0.001
Residence in OCV clusters: Better WASH households	24,307	16;29737	54 (27,80)	<0.001	53 (22,72)	0.004	48 (16,67)	0.007

* Person-years.

^¶^ Adjusted for closer distance from the household to the nearest icddr,b hospital, age at zero time (years), household average expenditure, individuals living in their own house, individuals living in a household with only one room, individuals living in a household with a concrete roof, individuals having reported diarrhoea within 6 months at the time of household registration, total number of household members.

# Protective effectiveness.

II One-sided p-value calculated using Cochran–Armitage test.

**Table 4:** Protection against all cholera and severe cholera episodes by receipt of 2 doses versus no OCV or by residence in Better or Not Better WASH households in the population evaluated for total OCV protection.

	Participants (n)	Case episodes;100000PY*	Incidence (Cases per 100,000 PY*; 95%CI)	p-value II (Trend test)	Crude PE# (95%CI)	p-value	Adjusted PE# (95%CI)	p-value
All cholera								
Residence in control clusters	78,518	222;105657	210 (183,238)		Ref.	–	Ref.	–
Receipt of OCV	61,970	97;79032	123 (98,147)	<0.001	41 (20,57)	0.001	40 (21,54) ƪ	<0.001
All cholera								
Residence in not Better WASH households	103,400	251;135602	185 (162,208)		Ref.	–	Ref.	–
Residence in Better WASH households	37,088	68;49087	139 (106,171)	0.033	25 (-1,45)	0.057	26 (0,46)	0.051
Severe cholera								
Residence in control clusters	78,518	105;105723	99 (80,118)		Ref.	–	Ref.	–
Receipt of OCV	61,970	34;79073	43 (29,57)	<0.001	57 (32,72)	<0.001	55 (32,70) ¶	<0.001
Severe cholera								
Residence in Not Better WASH households	103,400	116;135677	85 (70,101)		Ref.	–	Ref.	–
Residence in Better WASH households	37,088	23;49119	47 (28,66)	0.007	45 (20,63)	0.002	33 (5,53) §	0.024

* Person-years.

Adjusted for closer distance from the household to the nearest icddr,b hospital, age at zero time (years), household average expenditure, individuals living in a household with a concrete roof, individuals having reported diarrhea within 6 months at the time of household registration, the total number of household members, individuals household wash status, receipt of 2-doses of OCV.

# Protective effectiveness.

II One-sided p-value calculated using Cochran–Armitage test.

ƪ Adjusted for closer distance from the household to the nearest icddr,b hospital, age at zero time (years), household average expenditure, individuals living in a household with a concrete roof, individuals having reported diarrhea within 6 months at the time of household registration, total number of household members, individuals household wash status, household wash status.

¶ Adjusted for closer distance from the household to the nearest icddr,b hospital, age at zero time (years), household average expenditure, individuals living in their own house, individuals living in a household with only one room, individuals having reported diarrhoea within 6 months at the time of household registration, total number of household members, household wash status.

§ Adjusted for closer distance from the household to the nearest icddr,b hospital, age at zero time (years), household average expenditure, individuals living in their own house, individuals living in a household with only one room, individuals having reported diarrhoea within 6 months at the time of household registration, total number of household members, receipt of 2-doses of OCV.

**Table 5:** Protection against all cholera and severe cholera episodes by receipt of 2 doses versus no OCV and by residence in Better or Not Better WASH households considered conjointly in the population evaluated for total OCV protection.

	Participants (n)	Case episodes;100000PY*	Incidence (Cases per 100,000 PY*; 95%CI)	p-value II (Trend test)	Crude PE#	p-value	Adjusted PE#	p-valueΩ
All cholera								
Residence in control clusters: Not Better WASH households	56,983	178;76573	232 (198,267)		Ref.	–	Ref.	–
Residence in control clusters: Better WASH households	21,535	44;29084	151 (107,196)		35(5,56)	0.027	34(2,55) ¶	0.039
Residence in Not Better WASH households: receipt of OCV	46,417	73;59029	124 (95,152)		47(26,62)	<0.001	44(26,58) ¶	<0.001
Residence in Better WASH households: receipt of OCV	15,553	24;20003	120 (72,168)	<0.001	48(15,68)	0.010	49(21,67) ¶	0.002
Severe cholera								
Residence in control clusters: Not Better WASH households	56,983	89;76620	116 (92,140)		Ref.	–	Ref.	–
Residence in control clusters: Better WASH households	21,535	16;29103	55 (28,82)		53(29,69)	<0.001	39(13,58) §	0.003
Residence in Not Better WASH households: receipt of OCV	46,417	27;59057	46 (28,63)		61(36,76)	<0.001	57(35,72) §	<0.001
Residence in Better WASH households: receipt of OCV	15,553	7;20016	35 (9,61)	<0.001	70(35,86)	0.002	63(21,83) §	0.010

* Person-years.

# Protective effectiveness.

II One-sided p-value calculated using Cochran–Armitage test.

¶ Adjusted for closer distance from the household to the nearest icddr,b hospital, age at zero time (years), household average expenditure, individuals living in a household with a concrete roof, individuals having reported diarrhoea within 6 months at the time of household registration, total number of household members, individual’s household wash status.

§ Adjusted for closer distance from the household to the nearest icddr,b hospital, age at zero time (years), household average expenditure, individuals living in their own house, individuals living in a household with only one room, individuals having reported diarrhoea within 6 months at the time of household registration, total number of household members.

Ω Trend of adjusted PE is statistically significant at p-value < 0.05.

## 4 Discussion

Our analysis of the population considered for measurement of overall OCV protection revealed that a program of OCV vaccination, including both vaccinees and non-vaccinees in the population targeted for vaccination, and “Better WASH” preexisting in the households were independently associated with protection against all treated episodes of cholera as well as severe cholera, with protection against severe cholera higher than protection against all cholera episodes. However, when considered jointly, the combination of the OCV program and Better WASH in the household was not associated with greater protection than that for the OCV program among residents of “Not Better WASH” households or for residents of “Better WASH” households in clusters not assigned to OCV.

To probe further into these findings, we analyzed the interplay between actual receipt of OCV, rather than merely being targeted by the OCV program, and “Better WASH” in the household by limiting the analysis to the population evaluated for total OCV protection. In this analysis, receipt of two doses of OCV and “Better WASH” preexisting in the household were also independently associated with protection against cholera overall and severe cholera, with higher levels of protection against severe cholera than against cholera overall. However, in contrast to the evaluation of the program of OCV and preexisting WASH in the household, this analysis found a gradient of protection against overall and severe cholera, with non-vaccinees in “Not Better WASH” households at highest risk, and progressively greater protection in non-vaccinees in “Better WASH” households, to vaccinees in “Not Better WASH” households, to vaccinees residing in “Better WASH” households.

Our analysis had several strengths, including being based on a well-designed and conducted CRT of OCV, using a previously validated definition of “Better WASH” and “Not Better WASH”, and prospective and comprehensive, systematic surveillance for treated episodes of cholera, employing a prior definition of cholera and severe cholera. However, our analysis also had several important limitations. Although the analysis of overall OCV protection was randomized, for reasons described elsewhere [10] , it is likely that it underestimated overall protection, due to the influx of cholera into the clusters from the outside. Similarly, because the WASH variable was based on a simple set of ten questions administered at baseline and did not capture in detail the behaviors and practices responsible for protection by WASH against cholera, it is likely that the simple dichotomous variable for “Better WASH” and “Not Better WASH” used in our study also underestimated the protective impact of WASH against cholera. Moreover, although OCV was randomly assigned to clusters in this CRT, household WASH status was not. As well, because the WASH variable captured only WASH practices occurring naturally in the study population, the variable may not represent the predicted impact of externally imposed WASH interventions. Finally, the analysis of total OCV protection and WASH, which yielded findings more concordant with expectations about the OCV-WASH interplay, was based on a non-randomized comparison of vaccinees and non-vaccinees.

The literature available on the separate and combined effects of OCV and WASH is very limited. As mentioned earlier, the original purpose of this CRT was to assess whether a WASH intervention improved the protection conferred by OCV. The trial failed to find such a positive interaction between OCV and WASH but was limited by poor uptake of the WASH intervention [10]. A combined program of OCV and WASH undertaken in Haiti showed substantial protection against cholera, but the study was not designed to evaluate the separate and combined effects of OCV and WASH [13]. Several studies have modelled the interaction between OCV and WASH upon cholera, reporting that the combination might lead to faster extinction of cholera outbreaks than either intervention individually. However, these analyses were based on multiple assumptions and were not based on direct studies of the interaction of OCV and WASH in the field [14], [15].

Because of the limitations of our study and the divergent findings for the populations analyzed for overall and total OCV protection, we conclude that while our analysis suggests the potential for programs of OCV and WASH to act together to protect against cholera overall and against severe cholera, the analysis per se is insufficient to convincingly demonstrate a positive interaction. To address one limitation of our analysis, we plan to undertake further analysis of this CRT using the “fried egg” approach, which has been demonstrated to counter the bias caused by the ingress of cholera into the clusters from the outside [16]. Because the interaction between OCV and WASH in preventing cholera lies at the heart of the current WHO global initiative to dramatically reduce the global burden of endemic cholera, we encourage further, well-designed field studies on this topic. As it is no longer ethical to conduct randomized trials in which groups are randomly assigned to OCV versus no OCV, one possible future approach to better understand the incremental benefit of adding WASH to OCV would be to cluster-randomize groups to OCV plus a well-defined and formulated WASH intervention versus OCV only.

## Declaration of Competing Interest

The authors declare that they have no known competing financial interests or personal relationships that could have appeared to influence the work reported in this paper.

## Data Availability

Data will be made available on request.
