# Supplementary material for: The interplay between WASH practices and vaccination with oral cholera vaccines in protecting against cholera in urban Bangladesh: Reanalysis of a cluster-randomized trial
Source: Vaccine. 2023 Mar 31;41(14):2368–75. doi: 10.1016/j.vaccine.2023.02.054 (PMC10102718; doi:10.1016/j.vaccine.2023.02.054)
Supplement: Supplementary data 1 [file mmc1.docx]

**Supplementary Table 1: Baseline characteristics of the residence in “Better” and “Not better” WASH households of the trial**

|  | **Overall Protection** | | | **Total Protection** | | |
| --- | --- | --- | --- | --- | --- | --- |
|  | Residence in “Not Better” HH^¶^  (n=128,452) | Residence in “Better” HH^¶^  (n=46,279) | p-value | Residence in “Not Better” HH^¶^  (n=103,400) | Residence in “Better” HH^¶^  (n=37,088) | p-value |
| Mean age (SD*; years) | 23.9 ± 16.0 | 24.2 ± 15.5 | <0.001 | 23.8 ± 15.9 | 24.3 ± 15.4 | <0.001 |
| Male participants- n^#^ (%) | 62116 (48.4) | 22825 (49.3) | <0.001 | 48567 (47.0) | 17817 (48.0) | <0.001 |
| Live in own house- n^#^ (%) | 30939 (24.1) | 9028 (19.5) | <0.001 | 26265 (25.4) | 7453 (20.1) | <0.001 |
| Live in a household with only one room- n^#^ (%) | 107896 (84.0) | 34956 (75.5) | <0.001 | 86410 (83.6) | 27823 (75.0) | <0.001 |
| Live in a household with a concrete roof - n^#^ (%) | 115245 (89.7) | 35550 (76.8) | <0.001 | 92850 (89.8) | 28356 (76.5) | <0.001 |
| Diarrhoea within previous 6 months - n^#^ (%) | 17901 (13.9) | 5945 (12.8) | <0.001 | 15069 (14.6) | 4995 (13.5) | <0.001 |
| Mean time living in the area (SD*; months) | 72 ± 121.1 | 67.1 ± 113.4 | <0.001 | 76.1 ± 123.8 | 70.5 ± 116.2 | <0.001 |
| Lived in study area for less than 1 year- n^#^ (%) | 55308 (43.1) | 20290 (43.8) | 0.004 | 42196 (40.8) | 15601 (42.1) | <0.001 |
| Live in a household that knows about cholera vaccine- n^#^ (%) | 7236 (5.6) | 5140 (11.1) | <0.001 | 5022 (4.9) | 3618 (9.8) | <0.001 |
| Mean number of individuals per household (SD*) | 4.8 ± 2.0 | 4.6 ± 1.9 | <0.001 | 4.9 ± 1.9 | 4.6 ± 1.9 | <0.001 |
| Median distance to the nearest icddr,b hospital (IQR^ǁ^) | 1868 (1316,2479) | 1572 (811,2056) | <0.001 | 1858 (1297,2477) | 1571 (803,2049) | <0.001 |

* standard deviation

^#^ number of individuals

^ǁ^ interquartile range

^¶^Households
